# Glycan-binding preferences and genetic evolution of human seasonal influenza A(H3N2) viruses during 1999-2007 in Taiwan

**DOI:** 10.1371/journal.pone.0196727

**Published:** 2018-05-10

**Authors:** Ya-Fang Wang, Chuan-Fa Chang, Huey-Pin Tsai, Chia-Yu Chi, Ih-Jen Su, Jen-Ren Wang

**Affiliations:** 1 National Institute of Infectious Diseases and Vaccinology, National Health Research Institutes, Tainan, Taiwan; 2 Department of Medical Laboratory Science and Biotechnology, College of Medicine, National Cheng Kung University, Tainan, Taiwan; 3 Center of Infectious Disease and Signaling Research, National Cheng Kung University, Tainan, Taiwan; 4 Department of Pathology, National Cheng Kung University Hospital, Tainan, Taiwan; 5 Department of Pediatrics, National Cheng Kung University Hospital, Tainan, Taiwan; Consejo Superior de Investigaciones Cientificas, SPAIN

## Abstract

It is generally agreed that human influenza virus preferentially binds to α-2,6-linked sialic acid-containing receptors, and mutations that change the binding preference may alter virus infectivity and host tropism. Limited information is available on the glycan-binding specificity of epidemic influenza viruses. In this study, we systemically investigated the glycan-binding preferences of human influenza A(H3N2) viruses isolated from 1999 to 2007 in Taiwan using a high-throughput carbohydrate array. The binding patterns of 37 H3N2 viruses were classified into three groups with significant binding-pattern variations. The results showed that the carbohydrate-binding patterns of H3N2 varied over time. A phylogenetic analysis of the hemagglutinin gene also revealed progressive drift year to year. Of note, the viruses that caused large outbreaks in 1999 and 2003 showed glycan-binding preferences to both α-2,3 and α-2,6 sialylated glycans. Twenty amino acid substitutions were identified primarily at antigenic sites that might contribute to H3N2 virus evolution and the change in the glycan-binding patterns. This study provides not only a systematic analysis of the receptor-binding specificity of influenza clinical isolates but also information that could help to monitor the outbreak potential and virus evolution of influenza viruses.

## Introduction

Influenza A viruses cause major respiratory tract infections in humans and are responsible for annual seasonal influenza epidemics and occasional global pandemics. The influenza virus is a member of family *Orthomyxoviridae* and contains a segmented, negative-stranded RNA genome in an enveloped virion [1]. The influenza A virus caused three human pandemics in the 20th century: the Spanish flu of 1918 (H1N1), the Asian flu of 1957–1958 (H2N2), and the Hong Kong flu of 1967–1968 (H3N2). Among these subtypes, H1N1 and H3N2 continue to circulate in the human population, leading to annual epidemics. The H1N1 subtype is responsible for the fourth recorded influenza pandemic, A(H1N1)pdm09, which emerged from a strain previously sustained outside of the human population [2, 3].

The influenza A virus hemagglutinin (HA) is a homotrimeric glycoprotein that initiates infection by allowing the virus to attach to host cell sialic acid and mediates fusion of the viral and endosomal membranes [4]. HAs from different species differ in their receptor-binding specificities. It is generally accepted that human influenza viruses predominantly bind to sialic acid with α-2,6 linkages, whereas the HAs from avian influenza viruses predominantly recognize sialic acids with α-2,3 linkages to the underlying sugar. In addition, α-2,3- and α-2,6-linked sialic acid are heterogeneously distributed in the human respiratory tract. Sialic acid with α-2,6 linkages was dominant on epithelial cells in the upper human respiratory tract, while sialic acid with α-2,3 linkages was found in the lower human respiratory tract, such as non-ciliated bronchiolar cells and alveolar cells [3, 5]. Apart from the receptor-binding specificity, this HA-glycan interaction plays an important role in virus infection, tissue tropism, virulence, and interspecies transmission of influenza A viruses [6]. A switch in HA receptor specificity from α-2,3 to α-2,6 sialylated glycans could be imperative for the efficient transmission of these viruses [7]. However, previous studies have shown that the classification of the glycan-binding preferences of different HAs based on sialic acid linkage alone is insufficient to establish a correlation between HA receptor specificity and the efficient transmission of influenza A viruses [6]. Elucidating the characteristics that underline adaptation in humans in greater detail is currently possible using glycan arrays. HAs from a number of H1N1, H3N2, and H5N1 strains and their mutants have been analyzed on glycan arrays to assess binding to previously defined glycan structures [8–10].

Several studies have screened human H3N2 influenza viruses or recombinant HA proteins on the Consortium for Functional Glycomics (CFG) glycan array. H3N2 shows great diversity in the substructures bound by different strains in these studies [10–12]. In our previous study, we established a simple high-throughput platform to characterize the carbohydrate-binding preferences of influenza B viruses isolated from Taiwan and found that the glycan-binding preference was correlated with the HA genotype and clinical manifestations [13]. The receptor-binding preferences and evolution of human H3N2 clinical isolates have not been studied in detail. Therefore, we performed a comprehensive analysis of the glycan-binding profiles of human seasonal influenza A(H3N2) viruses isolated from 1999 to 2007 to explore the receptor-binding specificity and to correlate the epidemic potential with their carbohydrate-binding preferences. The patterns among glycan-binding preferences, genetic variations, phylogenetic evolution, and epidemiology over a nine-year period are discussed.

## Materials and methods

### Viruses and clinical cases

The human influenza H3N2 viruses used in this study were collected from the Virology Laboratory of National Cheng Kung University Hospital from 1999 to 2007. Throat and nasal swabs from patients with flu-like syndromes were collected by sentinel and National Cheng Kung University Hospital physicians regardless of age. Influenza A(H3N2) virus infection of cells was confirmed by the use of monoclonal antibodies for influenza A(H3N2) viruses (Chemicon, Inc.). We randomly selected 5–10 strains per year for glycan-binding analysis and sequencing.

### Amplification and purification of clinical influenza isolates

For virus preparation, stock viruses were propagated in Madin-Darby canine kidney (MDCK) cells (less than 4 passages) as previously described [14] and the vial titers of the clinical isolates were in the range of 64–512 HA units prior to concentration. For virus inactivation, a 100 μl stock of 5% merthiolate in PBS was added to 50 ml of the viral suspension to obtain a 1:10,000 final concentration of merthiolate and incubated at 4°C overnight [15]. The viral particles were then pelleted by ultracentrifugation in a 70 Ti rotor at 100,000 x g for 4 h purified through a 10–50% sucrose density gradient. The purified viral particles were re-suspended in PBS, and the protein concentrations were determined using the Bio-Rad protein assay (Bio-Rad Laboratories, Hercules, CA, USA).

### Glycan microarray analysis

**Fig 1:**
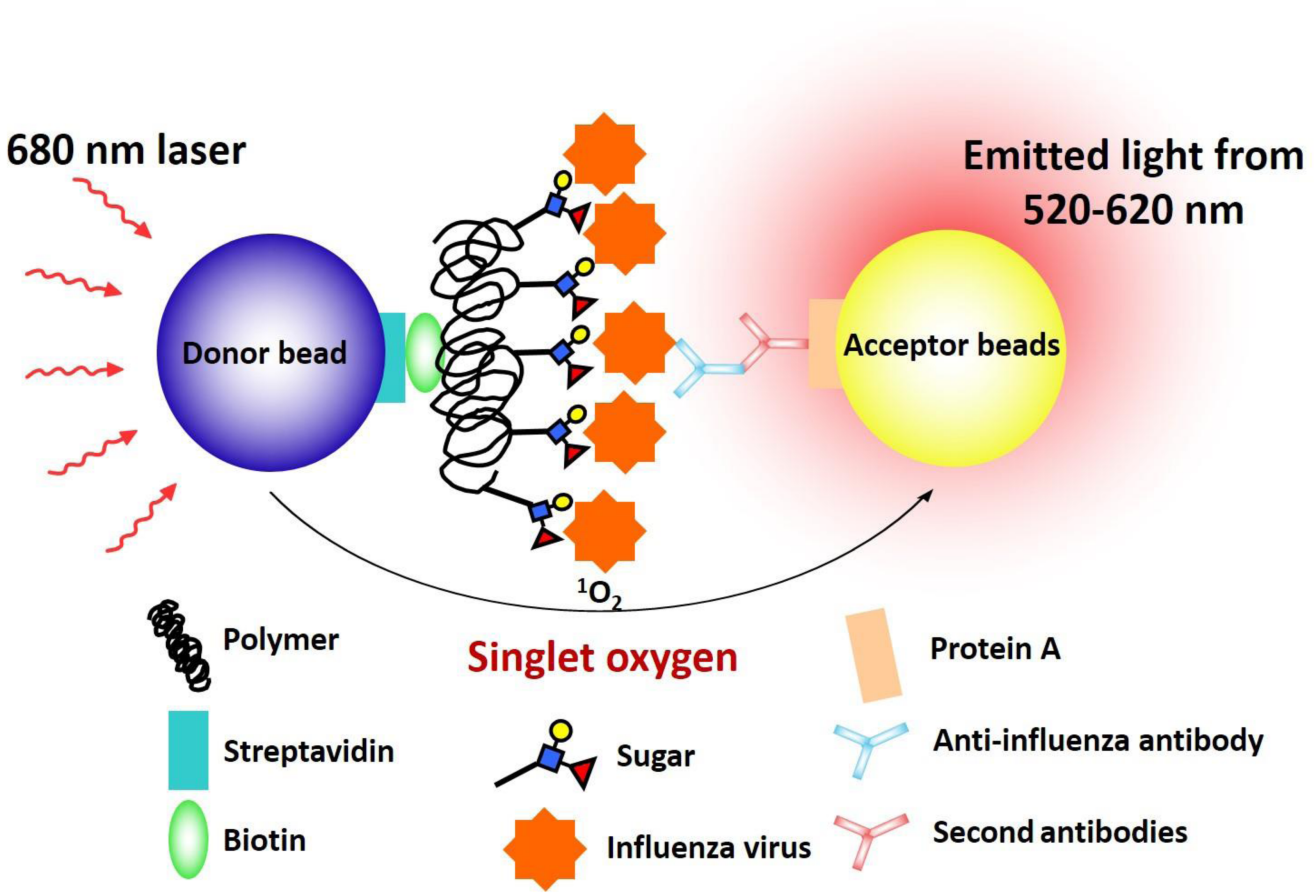
In-solution proximity binding with photosensitizers to characterize the glycan-binding preference of influenza viruses. This figure is modified from our previous publication [16]. Briefly, donor beads (500 ng/well) and biotin-PAA-sugars were mixed with inactivated viral particles. The mixture of acceptor beads, mouse anti-HA antibody, and rabbit anti-mouse IgG antibody was added. The binding signals were obtained on a PerkinElmer Envision instrument using the ALPHA Screen^TM^ program.

This platform is a non-washing, homogeneous solution carbohydrate array that uses polyacrylamide (PAA)-based glycans as previously described [16]. Briefly, donor beads (500 ng/well) and biotin-PAA-sugars (20 ng/well) (GlycoTech, Gaithersburg, MD, USA) were mixed with inactivated viral particles (20 ng/well for a total of 15 μl) and incubated for one hour. The mixture of acceptor beads (500 ng/well), mouse anti-HA antibody (30 ng/well) (Abcam, Cambridge, UK), and rabbit anti-mouse IgG antibody (25 ng/well) (Zymed, San Francisco, CA, USA) was added to a final volume of 25 μl (Fig 1). After 2 h of incubation, the binding signals were obtained on a PerkinElmer Envision instrument using the ALPHAScreen^TM^ program as previously described [16]. The results were presented as the relative strength of the highest fluorescence intensity in the same assay batch. A relative strength higher than 40% was considered a strong binding capacity and a relative strength lower than 20% was considered a weak interaction. As controls, the recombinant HA of H5N1 (Vietnam/1203/04) was added to wells in the same assay batch.

### Nucleotide-sequencing analysis

Viral RNA was extracted using a QIAamp kit. The primers and cycle conditions for RT-PCR and sequencing were previously described [14]. The nucleotide sequence of the purified DNA was determined using an automated DNA sequencer. A BigDye® Terminator v3.1 Cycle Sequencing Kit (ABI, Foster City, CA, USA) was used for sequencing with an ABI PRISM 3730XL Genetic Analyzer. DNA and amino acid sequence alignments were performed with the online software EBI ClustalW2 (http://www.ebi.ac.uk/Tools /clustalw/) and the BioEdit Sequence Alignment Editor version 7.0.5.3. A/Brisbane/10/2007 (GenBank accession number CY058075) was used as the reference sequence.

### Phylogenetic analysis

Phylogenetic comparisons were performed using version 3.573c of the Phylogeny Inference Package (PHYLIP) as previously described [14]. Consensus neighbor-joining trees were obtained from 1,000 bootstrap replicates of aligned HA1 sequences (amino acid positions 2 to 290) from different influenza H3N2 isolates. The branch length was estimated by the maximum likelihood method and bootstrap values >70% are indicated. Vaccine strains are indicated with an asterisk (*). The GenBank accession numbers for the vaccine strains are provided in brackets next to the virus name.

### Nucleotide sequence accession numbers

The influenza virus isolates used in this study were deposited in the GenBank database and assigned the accession numbers KU662097-KU662129 and MG309818-MG309872 (also see S1 Table).

## Results

### Glycan-binding profile of 37 human seasonal influenza A(H3N2) clinical isolates

**Fig 2:**
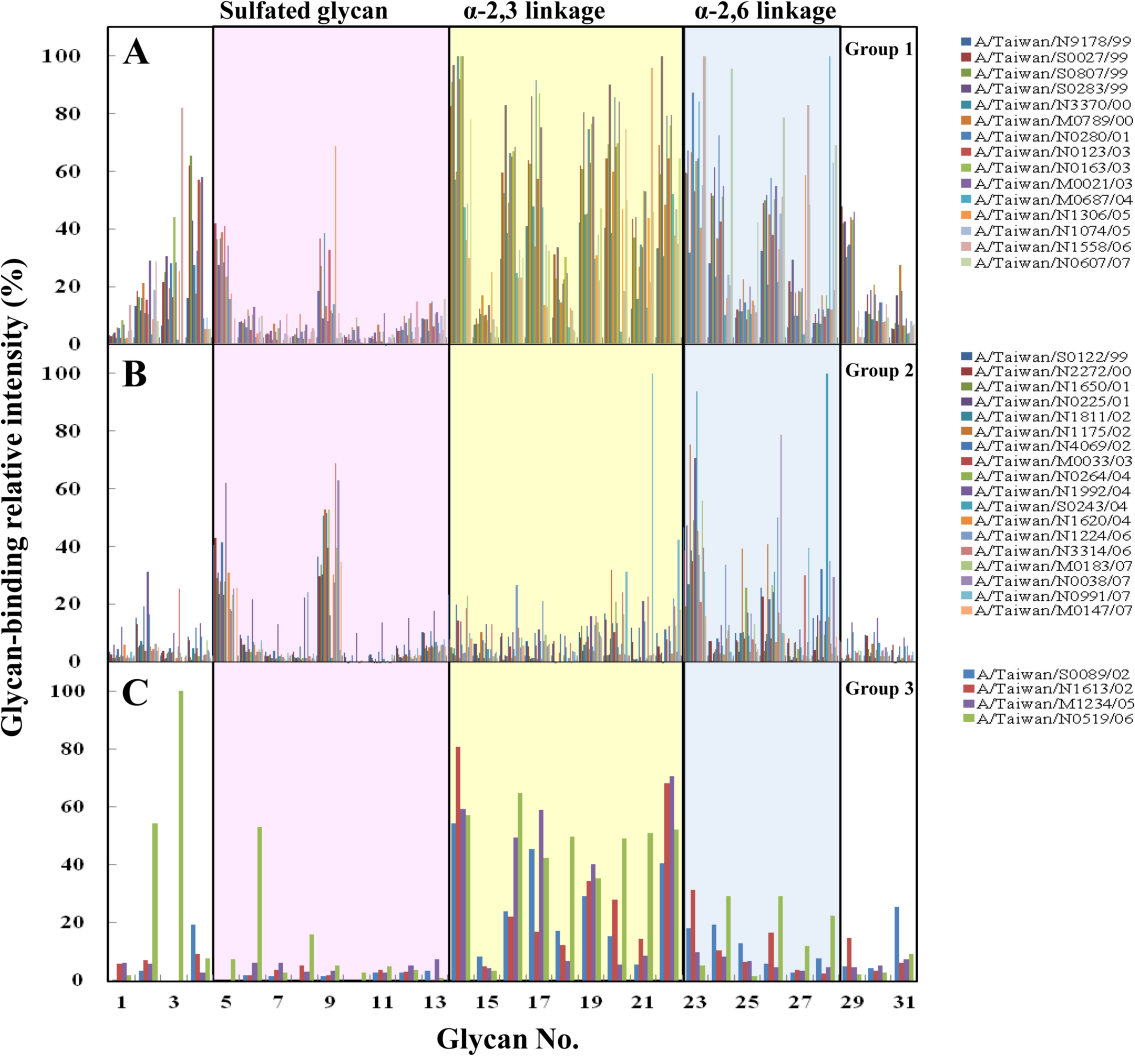
Glycan-binding profiles of 37 human seasonal influenza A(H3N2) clinical isolates. Glycan binding is indicated by the relative intensities of the highest fluorescence signal (*y* axis). The sugar identities are listed in Table 1 and are designated by numbers (*x* axis). The H3N2 clinical isolates were divided into 3 groups according to their binding preference. Group 1 viruses preferentially bound to both α-2,3 and α-2,6-linked sialylated glycans (A), group 2 viruses preferentially bound to α-2,6-linked sialylated glycans (B), and group 3 viruses specifically bound to α-2,3-linked sialylated glycans (C).

Thirty-seven H3N2 clinical isolates were successfully subjected to our glycan microarray analysis. The individual glycan structures are listed in Table 1. The binding profiles of whole virions to 30 sialoglycans on the glycan array allowed the categorization of the virus isolates into three groups (Fig 2). Group 1 viruses preferentially bound both to five α-2,6 sialylated glycans (NeuAcα2-6GalNAc, NeuAcα2-6Galβ, 6'sialyl-lactose, Galβ1-3(NeuAcα2–6)GalNAcα, and α2–6 sialylated diantennary N-glycans; glycans 23, 24, 26, 27, and 28) and seven α-2,3-linked sialylated glycans (NeuAcα2-3Gal, 3'sialyl-lactose, NeuAcα2-3Galβ1-4GlcNAcβ, NeuAcα2-3Galβ1-3GalNAcα, NeuAcα2-3(NeuAcα2–6)GalNAc, sialyl Le^a^, and sialyl Le^x^; glycans 14, 16, 17, 19, 20, 21, and 22; Fig 2A). Group 2 bound predominantly to three of the α-2,6-linked glycans (NeuAcα2-6GalNAc, 6'sialyl-lactose, and α2–6 sialylated diantennary N-glycans; glycans 23, 26, and 28; Fig 2B) but had weak interactions with NeuAcα2-6Galβ, NeuGcα2-6GalNAc, and Galβ1-3(NeuAcα2–6)GalNAcα(glycans 24, 25, and 27) and the α-2,3-linked sialylated glycans. In contrast to group 2, group 3 viruses had weak interactions with the α-2,6-linked sialylated glycans but specifically bound to six α-2,3-linked sialylated glycans (NeuAcα2-3Gal, 3'sialyl-lactose, NeuAcα2-3Galβ1-4GlcNAcβ, NeuAcα2-3Galβ1-3GalNAcα, NeuAcα2-3(NeuAcα2–6)GalNAc, and sialyl Le^x^; glycans 14, 16, 17, 19, 20, and 22; Fig 2C). Interestingly, although they belonged to the same H3N2 genotype, the group 1 isolates exhibited broader receptor-binding specificity than the group 2 and 3 viruses.

**Table 1:** Thirty glycans immobilized on polyacrylamide included in the glycan microarray analysis.

No.		PAA-Glycans	No.		PAA-Glycans
**1**		Blank-PAA-biotin	**17**	2–3 linkage	NeuAcα2-3Galβ1-4GlcNAcβ
**2**		α-NeuAc	**18**		3'Sialyl Le^c^
**3**		α-NeuAc-OCH_2_C_6_H_4_-*p*-NHCOOCH_2_	**19**		NeuAcα2-3Galβ1-3GalNAcα
**4**		α-NeuGc	**20**		NeuAcα2-3(NeuAcα2–6)GalNAc
**5**	Sulfated glycans	β-Gal-3-sulfate	**21**		Sialyl Le^a^
**6**		β-GlcNAc-6-sulfate	**22**		Sialyl Le^x^
**7**		3-HSO_3_-Galβ1-4GlcNAcβ	**23**	2–6 linkage	NeuAcα2-6GalNAc
**8**		3-HSO_3_-Galβ1-3GlcNAcβ	**24**		NeuAcα2-6Galβ
**9**		6-HSO_3_-Galβ1-4GlcNAcβ	**25**		NeuGcα2-6GalNAc
**10**		Galβ1-4(6-HSO_3_)GlcNAcβ	**26**		6'Sialyl-lactose
**11**		3-HSO_3_-Galβ1-3GalNAcβ (sulfate-TF)	**27**		Galβ1-3(NeuAcα2–6)GalNAcα
**12**		3-HSO_3_-Le^x^	**28**		α2–6 sialylated diantennary N-glycans
**13**		3-HSO_3_-Le^a^	**29**	2–8 linkage	NeuAcα2-8NeuAc
**14**	2–3 linkage	NeuAcα2-3Gal	**30**		NeuAcα2−8_5−6_
**15**		NeuAcα2-3GalNAcα	**31**		H_2_O
**16**		3'Sialyl-lactose			

No. 28: α2–6 sialylated diantennary N-glycans

(NeuAcα2-6Galβ1-4GlcNAcβ1-2Man)_2_ α1–3,6Manβ1-4GlcNAcβ1-4GlcNAc

Gal: galactose; GalNAc: *N*-acetylgalactosamine; Glc: glucose; GlcNAc: *N*-acetylglucosamine; NeuAc: *N*-acetylneuraminicacid; NeuGc: *N*-glycolylneuraminicacid.

### Patterns of glycan-binding preferences, epidemiology, and phylogenetic analysis

**Fig 3:**
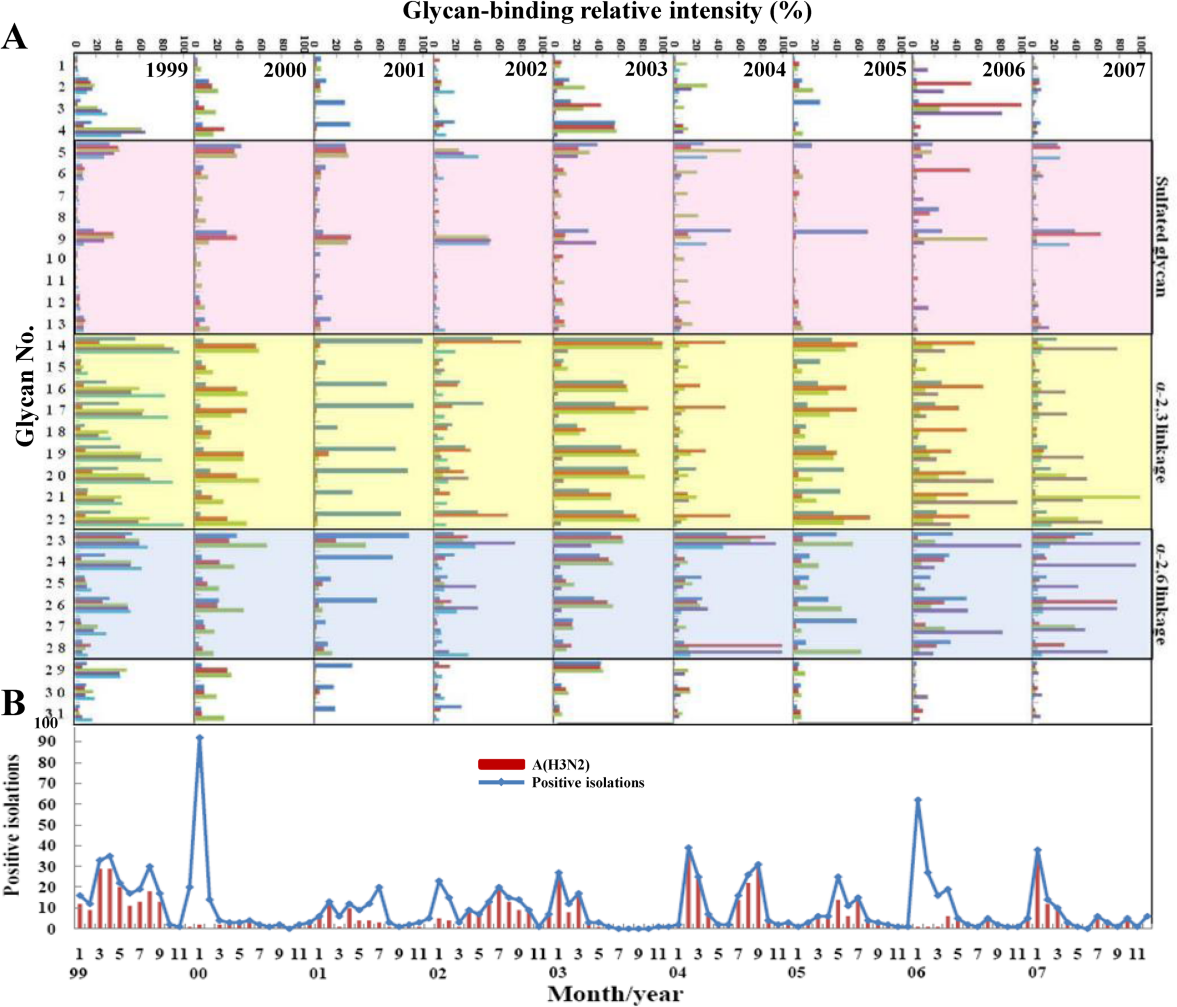
Pattern of the glycan-binding preference and epidemiology of human H3N2 viruses isolated from 1999 to 2007 in Taiwan. The glycan-binding patterns of the H3N2 clinical isolates are the same as shown in Fig 2 and are grouped by years (A) and combined with the monthly distribution of the positive isolates of the H3N2 virus counts and the total number of influenza virus-positive isolates from 1999 to 2007 in Taiwan (B).

To investigate whether the glycan-binding specificity of influenza A(H3N2) viruses correlated with epidemiology and virus evolution, the glycan-binding patterns of these H3N2 clinical isolates were grouped by years and compared to the monthly distribution of H3N2 isolate counts and the total number of influenza virus-positive isolates per month from 1999 to 2007 based on the collection by the Clinical Virology Laboratory of National Cheng Kung University Hospital. As shown in Fig 3, the viruses isolated in 1999 exhibited higher binding to both α-2,3 and α-2,6 sialylated glycans. The viruses then exhibited reduced affinity to both types of glycans until 2003, when the viruses re-acquired the binding affinity for both the α-2,3 and α-2,6 sialylated glycans. The viruses isolated in 2004 exhibited reduced binding affinity for the α-2,3 sialylated glycans compared to the viruses isolated in 2003.The ability to bind α-2,3 sialylated glycans was re-acquired in 2005 (Fig 3). These results indicated that the H3N2 viruses isolated from 1999 to 2007 demonstrated year-to-year variations in their receptor-binding specificities. Additionally, most of the H3N2 viruses isolated from 1999, 2003, and 2005 bound to both α-2,3 and α-2,6 sialylated glycans, whereas most of the viruses isolated from 2004 and 2007 bound predominantly to α-2,6 sialylated glycans. Interestingly, the viruses caused large outbreaks in 1999 and 2003 exhibited broad receptor-binding specificity (Fig 3).

**Fig 4:**
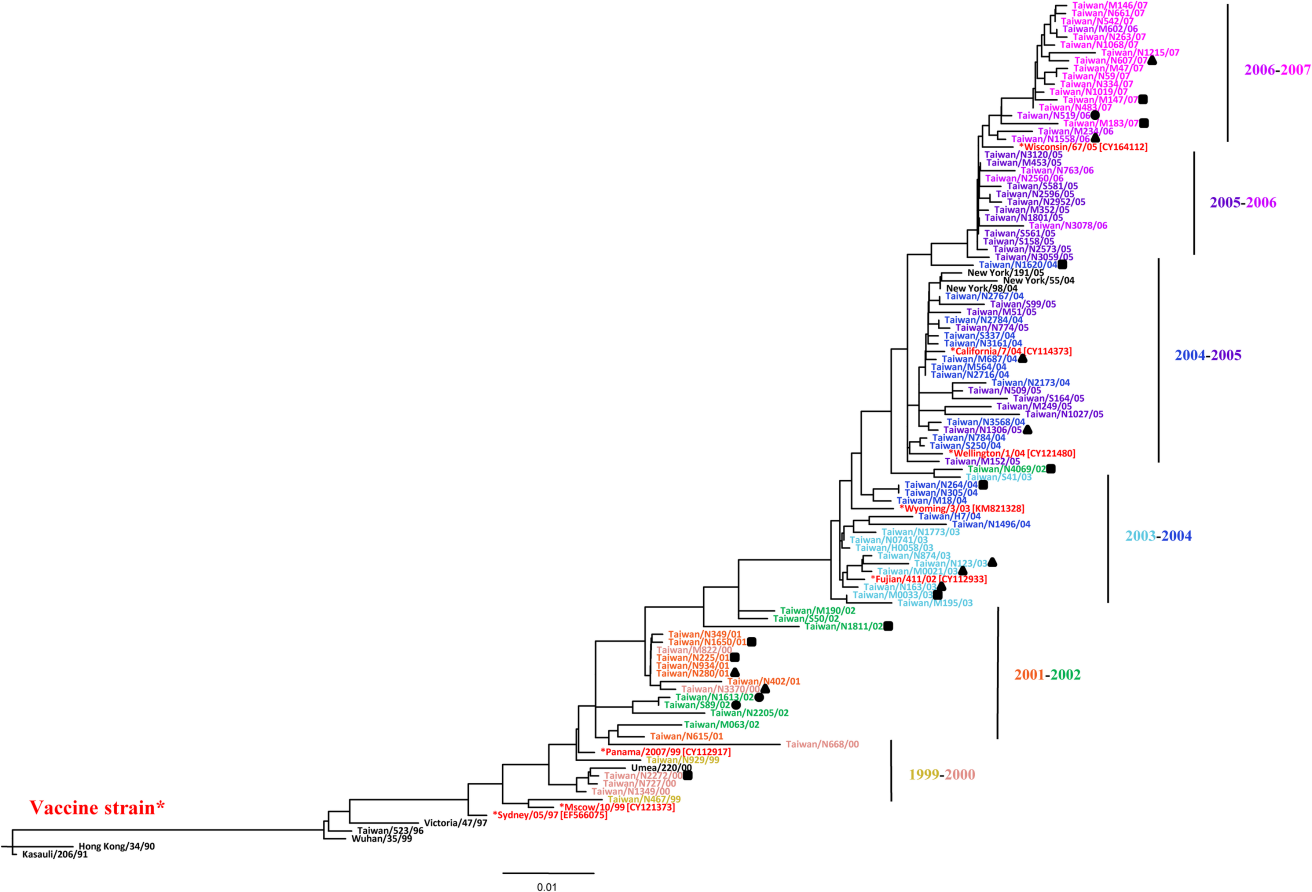
Phylogenetic analysis of human influenza A(H3N2) viruses isolated from 1999 to 2007in Taiwan. Consensus neighbor-joining trees were constructed from 1,000 bootstrap replicates of aligned HA1 sequences (nucleotide positions 52–915 of A/Brisbane/10/2007) from different influenza H3N2 isolates. The branch length was estimated by maximum likelihood and bootstrap values >70% are indicated at the nodes. *Asterisks indicate vaccine strains. Glycan-binding group 1 viruses are indicated with black triangles (▲), group 2 viruses are indicated with black circles (●), and group 3 viruses are indicated with black squares (■). The GenBank accession numbers for the vaccine strains are provided in brackets next to the virus names.

To explore H3N2’s genetic evolution, we examined the HA sequences (nucleotide positions 52–915 of A/Brisbane/10/2007) of the strains circulating in Taiwan from 1999 to 2007 by phylogenetic analysis. The phylogenic trees of the HA1 genes adopted a ladder-like structure with progressive drifts over time, revealing that continuous evolution occurred in the H3N2 isolates during this period (Fig 4). In addition, we also added the glycan-binding preferences of those that have been tested in carbohydrate array with different symbols into Fig 4. Although there were mixed glycan groups in the same year, the carbohydrate-binding patterns of H3N2 also revealed continuously changing patterns during the study period.

### Amino acid substitutions in HA sequences of H3N2 viruses

**Fig 5:**
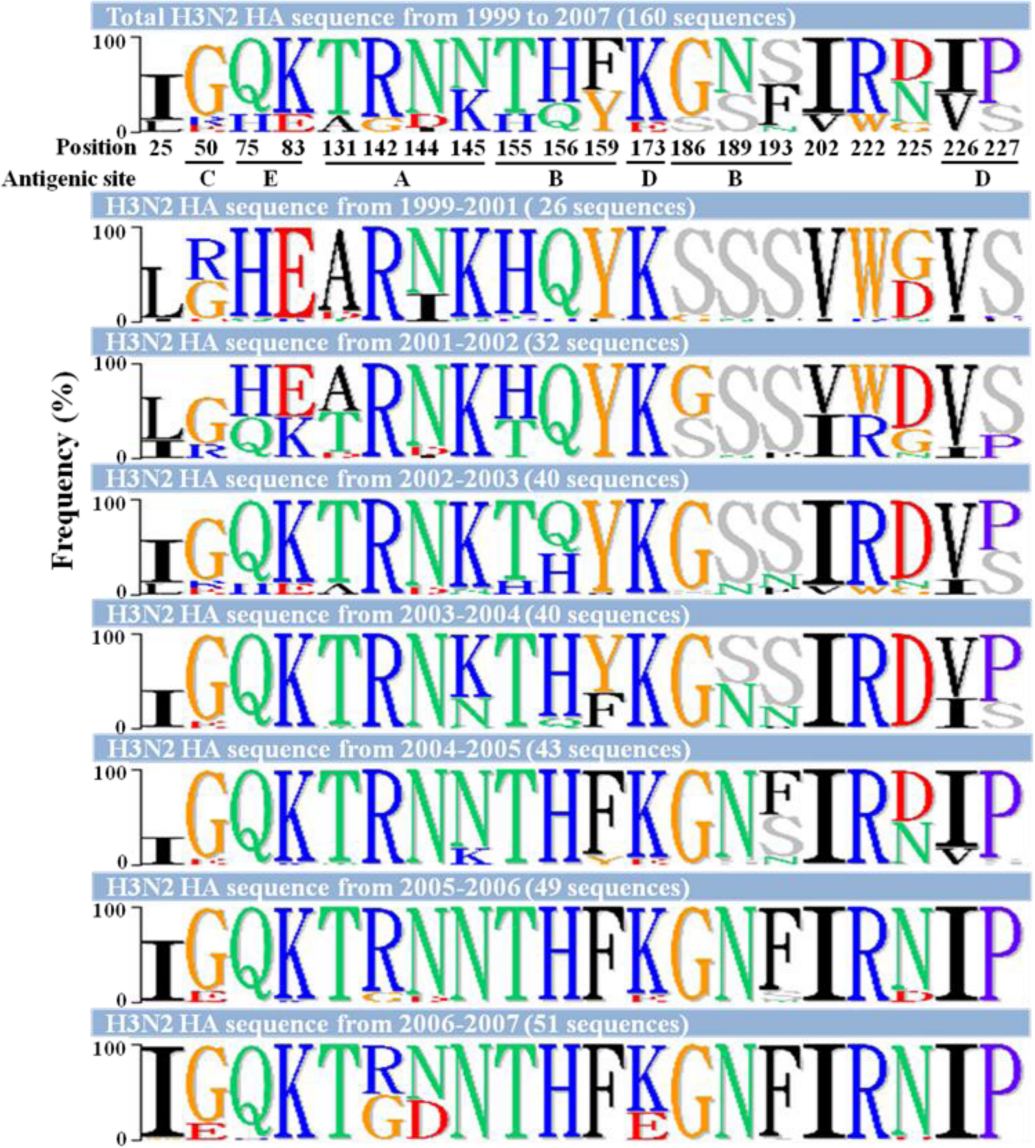
The frequency of amino acid substitutions in the HA1 gene of H3N2 viruses isolated from 1999 to 2007 in Taiwan. The amino acid sequences were aligned by the BioEdit Sequence Alignment program, and the gene signature was displayed using the Phylo-mLogo program. The frequency of amino acid sequences relative to the total number of sequences in each indicated period is shown.

To evaluate whether any amino acid substitutions contributed to the different glycan-binding preferences of the H3N2 influenza viruses, we aligned the viral HA sequences. The amino acid variations are shown in Table 2 with the antigenic sites indicated. The results showed that no specific sequences were associated with the α-2,3- or α-2,6-binding preferences of the H3N2 viruses. To analyze the accumulated substitutions involved in the evolution of the HA gene, we compared 160 available HA sequences from Taiwan human H3N2 strains collected from 1999 to 2007, including 126 strains retrieved from the GenBank database. The results showed that the greatest variation (six amino acid substitutions) was noted at antigenic site B, which was similar to previous observation [17] (Table 3 and Fig 5). Six waves of substitutions were observed when the HA1 gene sequences from 1999 to 2007 were compared. In 1999–2001, three substitutions occurred at R50G, I144N, and G225D. In 2001–2002, 10 substitutions occurred at L25I, H75Q, E83K, A131T, H155T, S186G, V202I, W222R, V226I, and S227P. In 2002–2003, two major additional substitutions occurred at Q156H and I226V. In 2003–2004, another five substitutions occurred at K145N, Y159F, S189N, V226I, and S227P. In 2004–2005, another two substitutions occurred at S193F and D225N. An additional three major substitutions (R142G, K173E, and N144D) appeared in 2005–2006 and 2006–2007. Interestingly, the glycan-binding patterns obviously changed over the years with the most amino acid substitutions (2001 to 2003 and 2003 to 2004) (Fig 3), especially at residues 193, 225, and 226, which have been reported to be important determinants for receptor-binding specificity [18, 19]. However, no specific sequence changes were correlated with different glycan-binding preferences. Identifying the dominant determinants for the glycan-binding preferences requires further investigation.

**Table 2:** Variations in HA amino acid sequences of influenza H3N2 viruses with different glycan-binding specificities.

	Amino acid sequence at position																				Glycan-binding group^a^
Antigenic site		C	E		A				B			D	B						D		
Virus strains	25	50	75	83	131	142	144	145	155	156	159	173	186	189	193	202	222	225	226	227	
**A/Brisbane/10/2007**^**b**^	**I**	**E**	**Q**	**K**	**T**	**R**	**N**	**N**	**T**	**H**	**F**	**K**	**G**	**N**	**F**	**I**	**R**	**N**	**I**	**P**	
**A/Taiwan/N3370/00**	**L**	**G**	**H**	**E**	**A**	**R**	**N**	**K**	**H**	**Q**	**Y**	**K**	**S**	**S**	**S**	**V**	**W**	**D**	**V**	**S**	**1**
**A/Taiwan/N0280/01**	**L**	**G**	**H**	**E**	**A**	**R**	**N**	**K**	**H**	**Q**	**Y**	**K**	**S**	**S**	**S**	**V**	**W**	**D**	**V**	**S**	**1**
**A/Taiwan/M0021/03**	**I**	**G**	**Q**	**K**	**T**	**R**	**N**	**K**	**T**	**H**	**Y**	**K**	**G**	**S**	**S**	**I**	**R**	**D**	**V**	**S**	**1**
**A/Taiwan/N0123/03**	**I**	**G**	**Q**	**K**	**T**	**R**	**N**	**K**	**T**	**H**	**Y**	**K**	**G**	**S**	**S**	**I**	**R**	**D**	**V**	**S**	**1**
**A/Taiwan/N0163/03**	**I**	**G**	**Q**	**K**	**T**	**R**	**N**	**Q**	**T**	**H**	**Y**	**K**	**G**	**S**	**S**	**I**	**R**	**D**	**V**	**P**	**1**
**A/Taiwan/M0687/04**	**I**	**G**	**Q**	**K**	**T**	**R**	**N**	**N**	**T**	**H**	**F**	**K**	**G**	**N**	**S**	**I**	**R**	**D**	**I**	**P**	**1**
**A/Taiwan/N1306/05**	**I**	**G**	**Q**	**K**	**T**	**R**	**N**	**N**	**T**	**H**	**F**	**K**	**G**	**N**	**S**	**I**	**R**	**D**	**I**	**P**	**1**
**A/Taiwan/N1558/06**	**I**	**G**	**Q**	**K**	**T**	**G**	**N**	**N**	**T**	**H**	**F**	**K**	**G**	**N**	**F**	**I**	**R**	**N**	**I**	**P**	**1**
**A/Taiwan/N0607/07**	**I**	**G**	**Q**	**K**	**T**	**G**	**D**	**N**	**T**	**H**	**F**	**E**	**G**	**N**	**F**	**I**	**R**	**N**	**I**	**P**	**1**
**A/Taiwan/N2272/00**	**L**	**R**	**H**	**E**	**A**	**R**	**I**	**K**	**H**	**Q**	**Y**	**K**	**S**	**S**	**S**	**V**	**W**	**G**	**V**	**S**	**2**
**A/Taiwan/N1650/01**	**L**	**G**	**H**	**E**	**A**	**R**	**N**	**K**	**H**	**Q**	**Y**	**K**	**S**	**S**	**S**	**V**	**W**	**G**	**V**	**S**	**2**
**A/Taiwan/N0225/01**	**L**	**G**	**H**	**E**	**A**	**R**	**N**	**K**	**H**	**Q**	**Y**	**K**	**S**	**S**	**S**	**V**	**W**	**D**	**V**	**S**	**2**
**A/Taiwan/N1811/02**	**L**	**R**	**H**	**E**	**A**	**R**	**D**	**K**	**H**	**Q**	**Y**	**K**	**G**	**S**	**F**	**I**	**R**	**N**	**I**	**P**	**2**
**A/Taiwan/N4069/02**	**I**	**G**	**Q**	**K**	**T**	**R**	**N**	**K**	**T**	**Q**	**Y**	**K**	**G**	**N**	**F**	**I**	**R**	**N**	**I**	**P**	**2**
**A/Taiwan/M0033/03**	**I**	**G**	**Q**	**K**	**T**	**R**	**N**	**K**	**T**	**Q**	**Y**	**K**	**G**	**S**	**S**	**I**	**R**	**D**	**V**	**P**	**2**
**A/Taiwan/N0264/04**	**I**	**G**	**Q**	**K**	**T**	**R**	**N**	**K**	**T**	**H**	**Y**	**K**	**G**	**S**	**N**	**I**	**R**	**D**	**I**	**P**	**2**
**A/Taiwan/N1620/04**	**I**	**G**	**Q**	**K**	**T**	**R**	**N**	**K**	**T**	**H**	**F**	**K**	**G**	**N**	**F**	**I**	**R**	**D**	**V**	**P**	**2**
**A/Taiwan/M0147/07**	**I**	**G**	**Q**	**K**	**T**	**G**	**D**	**N**	**T**	**H**	**F**	**E**	**G**	**N**	**F**	**I**	**R**	**N**	**I**	**P**	**2**
**A/Taiwan/M0183/07**	**I**	**G**	**Q**	**K**	**T**	**G**	**N**	**N**	**T**	**H**	**F**	**E**	**G**	**N**	**F**	**I**	**R**	**N**	**I**	**P**	**2**
**A/Taiwan/S0089/02**	**L**	**G**	**H**	**E**	**A**	**R**	**N**	**K**	**H**	**Q**	**Y**	**K**	**G**	**S**	**S**	**I**	**R**	**N**	**I**	**P**	**3**
**A/Taiwan/N1613/02**	**L**	**R**	**H**	**E**	**A**	**R**	**D**	**K**	**H**	**Q**	**Y**	**K**	**G**	**S**	**S**	**V**	**W**	**G**	**V**	**S**	**3**
**A/Taiwan/N0519/06**	**I**	**G**	**Q**	**K**	**T**	**G**	**N**	**N**	**T**	**H**	**F**	**K**	**G**	**N**	**F**	**I**	**R**	**N**	**I**	**P**	**3**

^a^Group 1 viruses were preferentially bound to both α-2,3- and α-2,6-linked sialylated glycans, and group 2 viruses were preferentially bound to α-2,6-linked sialylated glycans and group 3 viruses bound specifically to α-2,3-linked sialyl glycans.

^b^Reference strain.

**Table 3:** Amino acid substitution patterns of H3N2 viruses isolated from 1999 to 2007 in Taiwan.

	Amino acid substitution																			
Antigenic site		C	E		A				B			D	B						D	
Virus strains	25	50	75	83	131	142	144	145	155	156	159	173	186	189	193	202	222	225	226	227
**A/Sydney/5/1997**	**L**	**R**	**H**	**E**	**A**	**S**	**I**	**K**	**H**	**Q**	**Y**	**K**	**S**	**S**	**S**	**V**	**W**	**G**	**I**	**S**
**A/Panama/2007/99**	**L**	**R**	**H**	**E**	**A**	**R**	**N**	**N**	**H**	**Q**	**Y**	**K**	**S**	**S**	**S**	**V**	**W**	**G**	**V**	**S**
**A/Moscow/10/99**	**L**	**R**	**H**	**E**	**A**	**R**	**I**	**N**	**H**	**Q**	**Y**	**K**	**S**	**S**	**S**	**V**	**W**	**G**	**I**	**S**
**A/Fujian/411/02**	**I**	**G**	**Q**	**K**	**T**	**R**	**N**	**K**	**T**	**H**	**Y**	**K**	**G**	**S**	**S**	**I**	**R**	**G**	**V**	**S**
**A/Wellington/01/04**	**I**	**G**	**Q**	**K**	**T**	**R**	**N**	**K**	**T**	**H**	**F**	**K**	**G**	**N**	**N**	**I**	**R**	**D**	**V**	**P**
**A/California/7/04**	**I**	**G**	**Q**	**K**	**T**	**R**	**N**	**N**	**T**	**H**	**F**	**K**	**G**	**N**	**S**	**I**	**R**	**D**	**I**	**P**
**A/Wisconsin/67/05**	**I**	**G**	**Q**	**K**	**T**	**R**	**N**	**N**	**T**	**Q**	**F**	**K**	**V**	**N**	**F**	**I**	**R**	**N**	**I**	**P**
**A/Brisbane/10/07**	**I**	**E**	**Q**	**K**	**T**	**R**	**N**	**N**	**T**	**H**	**F**	**K**	**G**	**N**	**F**	**I**	**R**	**N**	**I**	**P**
**A/Taiwan/N0467/99**	**L**	**R**	**H**	**E**	**A**	**R**	**I**	**K**	**P**	**Q**	**Y**	**K**	**S**	**S**	**S**	**V**	**W**	**G**	**V**	**S**
**A/Taiwan/N0929/99**	**L**	**R**	**H**	**E**	**A**	**R**	**N**	**K**	**H**	**Q**	**Y**	**K**	**S**	**S**	**S**	**V**	**W**	**G**	**V**	**S**
**A/Taiwan/M0822/00**	**L**	**G**	**H**	**E**	**A**	**R**	**N**	**K**	**H**	**Q**	**Y**	**K**	**S**	**S**	**S**	**V**	**W**	**D**	**V**	**S**
**A/Taiwan/N0668/00**	**L**	**R**	**H**	**E**	**A**	**R**	**N**	**K**	**H**	**Q**	**Y**	**K**	**S**	**S**	**S**	**V**	**W**	**G**	**V**	**S**
**A/Taiwan/N0727/00**	**L**	**R**	**H**	**E**	**A**	**R**	**I**	**K**	**H**	**Q**	**Y**	**K**	**S**	**S**	**S**	**V**	**W**	**G**	**V**	**S**
**A/Taiwan/N0225/01**	**L**	**G**	**H**	**E**	**A**	**R**	**N**	**K**	**H**	**Q**	**Y**	**K**	**S**	**S**	**S**	**V**	**W**	**D**	**V**	**S**
**A/Taiwan/N0615/01**	**L**	**G**	**H**	**E**	**A**	**R**	**N**	**K**	**H**	**Q**	**Y**	**K**	**S**	**S**	**S**	**V**	**W**	**G**	**V**	**S**
**A/Taiwan/N0934/01**	**L**	**G**	**H**	**E**	**A**	**R**	**N**	**K**	**H**	**Q**	**Y**	**K**	**S**	**S**	**S**	**V**	**W**	**D**	**V**	**S**
**A/Taiwan/M0063/02**	**L**	**G**	**H**	**E**	**A**	**R**	**N**	**K**	**H**	**Q**	**Y**	**K**	**S**	**S**	**S**	**V**	**W**	**G**	**V**	**S**
**A/Taiwan/N2205/02**	**L**	**R**	**H**	**E**	**A**	**R**	**D**	**K**	**H**	**Q**	**Y**	**K**	**G**	**S**	**S**	**V**	**W**	**G**	**V**	**S**
**A/Taiwan/N1811/02**	**L**	**R**	**H**	**E**	**A**	**R**	**D**	**K**	**H**	**Q**	**Y**	**K**	**G**	**S**	**F**	**I**	**R**	**N**	**I**	**P**
**A/Taiwan/N4069/02**	**I**	**G**	**Q**	**K**	**T**	**R**	**N**	**K**	**T**	**Q**	**Y**	**K**	**G**	**N**	**F**	**I**	**R**	**N**	**I**	**P**
**A/Taiwan/M0033/03**	**I**	**G**	**Q**	**K**	**T**	**R**	**N**	**K**	**T**	**Q**	**Y**	**K**	**G**	**S**	**S**	**I**	**R**	**D**	**V**	**P**
**A/Taiwan/M0195/03**	**I**	**G**	**Q**	**K**	**T**	**R**	**N**	**K**	**T**	**Q**	**Y**	**K**	**G**	**S**	**S**	**I**	**R**	**D**	**V**	**P**
**A/Taiwan/M0021/03**	**I**	**G**	**Q**	**K**	**T**	**R**	**N**	**K**	**T**	**H**	**Y**	**K**	**G**	**S**	**S**	**I**	**R**	**D**	**V**	**S**
**A/Taiwan/N0123/03**	**I**	**G**	**Q**	**K**	**T**	**R**	**N**	**K**	**T**	**H**	**Y**	**K**	**G**	**S**	**S**	**I**	**R**	**D**	**V**	**S**
**A/Taiwan/N0874/03**	**I**	**G**	**Q**	**K**	**T**	**R**	**N**	**K**	**T**	**H**	**Y**	**K**	**G**	**S**	**S**	**I**	**R**	**D**	**V**	**S**
**A/Taiwan/N0163/03**	**I**	**G**	**Q**	**K**	**T**	**R**	**N**	**Q**	**T**	**H**	**Y**	**K**	**G**	**S**	**S**	**I**	**R**	**D**	**V**	**P**
**A/Taiwan/N0264/04**	**I**	**G**	**Q**	**K**	**T**	**R**	**N**	**K**	**T**	**H**	**Y**	**K**	**G**	**S**	**N**	**I**	**R**	**D**	**I**	**P**
**A/Taiwan/N1620/04**	**I**	**G**	**Q**	**K**	**T**	**R**	**N**	**K**	**T**	**H**	**F**	**K**	**G**	**N**	**F**	**I**	**R**	**D**	**V**	**P**
**A/Taiwan/S0337/04**	**I**	**G**	**Q**	**K**	**T**	**R**	**N**	**N**	**T**	**H**	**F**	**K**	**G**	**N**	**S**	**I**	**R**	**D**	**I**	**P**
**A/Taiwan/M0687/04**	**I**	**G**	**Q**	**K**	**T**	**R**	**N**	**N**	**T**	**H**	**F**	**K**	**G**	**N**	**S**	**I**	**R**	**D**	**I**	**P**
**A/Taiwan/N1306/05**	**I**	**G**	**Q**	**K**	**T**	**R**	**N**	**N**	**T**	**H**	**F**	**K**	**G**	**N**	**S**	**I**	**R**	**D**	**I**	**P**
**A/Taiwan/N1801/05**	**I**	**G**	**Q**	**K**	**T**	**R**	**N**	**N**	**T**	**H**	**F**	**K**	**G**	**N**	**F**	**I**	**R**	**N**	**I**	**P**
**A/Taiwan/N2573/05**	**I**	**G**	**Q**	**K**	**T**	**R**	**N**	**N**	**T**	**H**	**F**	**K**	**G**	**N**	**F**	**I**	**R**	**N**	**I**	**P**
**A/Taiwan/N2952/05**	**I**	**G**	**Q**	**K**	**T**	**R**	**N**	**N**	**T**	**H**	**F**	**K**	**G**	**N**	**F**	**I**	**R**	**N**	**I**	**P**
**A/Taiwan/N2560/06**	**I**	**G**	**Q**	**K**	**T**	**R**	**N**	**N**	**T**	**H**	**F**	**K**	**G**	**N**	**F**	**I**	**R**	**N**	**I**	**P**
**A/Taiwan/N0519/06**	**I**	**G**	**Q**	**K**	**T**	**G**	**N**	**N**	**T**	**H**	**F**	**K**	**G**	**N**	**F**	**I**	**R**	**N**	**I**	**P**
**A/Taiwan/N1558/06**	**I**	**G**	**Q**	**K**	**T**	**G**	**N**	**N**	**T**	**H**	**F**	**K**	**G**	**N**	**F**	**I**	**R**	**N**	**I**	**P**
**A/Taiwan/M0602/06**	**I**	**G**	**Q**	**K**	**T**	**G**	**D**	**N**	**T**	**H**	**F**	**E**	**G**	**N**	**F**	**I**	**R**	**N**	**I**	**P**
**A/Taiwan/M0183/07**	**I**	**G**	**Q**	**K**	**T**	**G**	**N**	**N**	**T**	**H**	**F**	**E**	**G**	**N**	**F**	**I**	**R**	**N**	**I**	**P**
**A/Taiwan/M0147/07**	**I**	**G**	**Q**	**K**	**T**	**G**	**D**	**N**	**T**	**H**	**F**	**E**	**G**	**N**	**F**	**I**	**R**	**N**	**I**	**P**
**A/Taiwan/N0607/07**	**I**	**G**	**Q**	**K**	**T**	**G**	**D**	**N**	**T**	**H**	**F**	**E**	**G**	**N**	**F**	**I**	**R**	**N**	**I**	**P**
**A/Taiwan/N0661/07**	**I**	**G**	**Q**	**K**	**T**	**G**	**D**	**N**	**T**	**H**	**F**	**E**	**G**	**N**	**F**	**I**	**R**	**N**	**I**	**P**

## Discussion

The binding of the influenza viral HA protein to the sialic acid-containing receptors on the surface of host cells is the key first step to initiate viral entry and infection. This study performed a systematic investigation of the glycan-binding biology of HA using human seasonal influenza A(H3N2) clinical isolates from Taiwan over a nine-year time period based on the glycan array. The binding patterns were successfully profiled for all studied viruses. Each virus exhibited a different and clear pattern, thereby enabling characterization. The H3N2 viruses isolated from 1999 to 2007 were classified into three groups based on their binding patterns. The data also revealed that the changes in the carbohydrate-binding patterns of the H3N2 viruses varied over time, which was similar to the phylogenetic analysis patterns. Although the specific HA amino acid sequences that might contribute to the different glycan-binding preferences were not identified, some amino acid variants located at antigenic sites or near the receptor-binding sites of H3N2 were observed. To the best of our knowledge, this is the first report to systematically analyze the glycan-binding biology of clinical human influenza A(H3N2) viruses circulating in Taiwan.

Influenza viruses infect approximately 5% to 15% of the global population, resulting in an estimated 3–5 million hospitalizations and approximately 500,000 deaths annually [20, 21]. The influenza A(H3N2) virus is currently the major cause of human influenza morbidity and mortality worldwide and shows the strongest antigenic drift [22, 23]. The receptor-binding specificity of H3N2 influenza viruses has been studied by several researchers. For example, Kumari et al. used fluorescently labeled virions to identify potential receptors for human H3N2 viruses with different abilities to agglutinate chicken erythrocytes using the Consortium for Functional Glycomics (CFG) glycan array. The results revealed that all recent H3N2 viruses exclusively bound to a subset of α-2,6 sialysaccharides [10]. Other researchers also examined human H3N2 viruses or recombinant HAs using the same platform. These studies observed that the binding patterns of different isolates had substantial diversity in their glycan substructures [11, 24, 25]. Our results were in agreement with these reports and also showed diverse variations between different isolates, with some isolates specifically binding to α-2,3 sialylated glycans. Moreover, previous studies indicated that H3N2 human isolates lost their ability to bind to the avian receptor in the 1990s, although viruses isolated since 2003 regained the ability to agglutinate chicken erythrocytes [10, 26]. Some studies demonstrated that viruses with a reduced ability to bind to both the human and avian receptors exhibited impaired growth in eggs and different cells in culture [25, 27]. In our study, we also observed that viruses isolated in 1999 bound to both α-2,3 and α-2,6 sialylated glycans, whereas viruses isolated in 2001 and 2002 showed a reduced binding affinity for α-2,3 sialylated glycans. The viruses isolated from 2003 re-acquired the ability to bind to glycans with α-2,3 linkages. The viruses isolated in 2004 exhibited reduced binding affinity for α-2,3 sialylated glycans again and re-acquired the ability to bind to α-2,3 sialylated glycans in 2005. Determining whether viruses with different affinities for α-2,3 and α-2,6 sialylated glycans exhibit differences in growth in eggs and cell cultures requires further investigation.

The development of glycan arrays in the early 2000s revolutionized influenza virus receptor-binding specificity research [28]. The glycan array is a good tool to investigate how HA-binding preferences of influenza viruses vary for the increasingly specific types of sialic acids. The glycan microarray is also a powerful tool to examine carbohydrate-protein interactions and provides a new platform for the characterization of influenza viruses by subtyping [29]. The glycan array used in this study was a solution array that was performed in a homogeneous solution, thereby avoiding the loss of weak binding partners during the repeated washes of the glycan microarrays. However, antigen/ligand excess effect may occur in this solution assay if the concentrations of proteins or antibodies are too high. To improve the binding signals between carbohydrates with protein/virus, we used polyacrylamide-backboned oligosaccharide substrates in this assay to enhance the multivalence. In addition, radical scavengers in reaction mixture may reduce the signal and result in false-negative results [30]. However, this method could provide good sensitivity (i.e., femtomole detection under optimized conditions) by relying on the binding affinity between analyses. Our previous study had demonstrated that the solution carbohydrate array provides a useful tool that is comparable to the other methods [16]. A previous study used CFG arrays to examine the binding properties of the major variants of human H3N2 viruses from 1968 to 2012 and found that the viruses isolated from 1997 to 2008 bound strongly to long polylactosamine chains terminating in sialic acid and had lost their ability to bind short branched α-2,6 sialylated glycans [12]. This phenomenon was not clearly observed in our study. Therefore, the binding preference of H3N2 influenza viruses in this study was different from other studies possibly due to the use of different detection methods.

The influenza virus undergoes rapid evolution in nature by both genetic shift, where one (or more) of the eight gene segments is exchanged from one virus to another [31], and genetic drift, whereby mutations accumulate in viral genes [32] presumably due to the relatively error-prone replication of the viral RNA [33].The antigenic shifts continuously change every two to three years. In this present study, we explored whether H3N2 evolution affects the glycan-binding patterns. The results indicated that the year-to-year variation and continuously changing receptor-binding specificity patterns of the H3N2 viruses were similar to the phylogenetic analysis results. Additionally, the binding preferences of H3N2 viruses collected in 1999 and 2003 were more consistent between different isolates than the viruses collected in the other years, and we also noted that these viruses caused large outbreaks in Taiwan showed glycan-binding preference to both α-2,6 and α-2,3 sialylated glycans. Previous studies showed that the various binding preferences of H3N2 viruses had no apparent consequences for disease or spread [12], and our research revealed similar results that the glycan-binding preferences did not significantly correlate with the epidemiology. Although the specific sequences associated with different binding preferences were not identified, we observed obvious changes in the glycan-binding patterns with increased amino acid substitutions in 2001–2003 and 2003–2004. Furthermore, the changing glycan-binding preference patterns of the viruses isolated in 1999–2001, 2003–2004, and 2004–2005 were simultaneously accompanied by substitutions at resides 193, 225, and 226, which closed the receptor-binding site at the 190-helix and 220-loop of HA1. These three amino acids have been reported to be important residues for determining receptor specificity [18, 19]. Because the antigenic differences and changes in the sialic acid receptor-binding properties of HA play important roles in the mechanisms underlying influenza virus evolution, identifying the dominant determinants for the glycan-binding preferences warrants further investigation.

In conclusion, we successfully examined the glycan-binding specificities beyond the α-2,6 and α-2,3 linkages of influenza A viruses using a glycan solution array platform. We also demonstrated that the glycan-binding patterns of human H3N2 changed year to year and the viruses that exhibited broad receptor-binding specificity appeared to relate to large outbreaks in 1999 and 2003, but the correlation remains to be determined. Future influenza pandemics seem inevitable, and predicting the potential HA subtypes that will emerge is a challenging task [34]. This study provides a systematic analysis of the receptor-binding specificities of influenza clinical isolates and information that could help monitor the pandemic potential and evolution of influenza viruses.

## Supporting information

S1 Table The list of the influenza viruses accession numbers used in this study. (PDF)

## References

[1] Kobayashi Y, Suzuki Y. Evidence for N-glycan shielding of antigenic sites during evolution of human influenza A virus hemagglutinin. Journal of virology. 2012;86(7):3446–51. doi: 10.1128/JVI.06147-11 . 22258255 10.1128/JVI.06147-11 PMC3302543

[2] Viswanathan K, Koh X, Chandrasekaran A, Pappas C, Raman R, Srinivasan A, et al Determinants of glycan receptor specificity of H2N2 influenza A virus hemagglutinin. PloS one. 2010;5(10):e13768 doi: 10.1371/journal.pone.0013768 . 21060797 10.1371/journal.pone.0013768 PMC2966429

[3] Wilks S, de Graaf M, Smith DJ, Burke DF. A review of influenza haemagglutinin receptor binding as it relates to pandemic properties. Vaccine. 2012;30(29):4369–76. doi: 10.1016/j.vaccine.2012.02.076 . 22682293 10.1016/j.vaccine.2012.02.076 PMC3372863

[4] Skehel JJ, Wiley DC. Receptor binding and membrane fusion in virus entry: the influenza hemagglutinin. Annual review of biochemistry. 2000;69:531–69. doi: 10.1146/annurev.biochem.69.1.531 . 10966468 10.1146/annurev.biochem.69.1.531

[5] Kyoko Shinya ME, Yamada Shinya, Ono Masao, Kasai Noriyuki, Kawaoka Yoshihiro. Avian flu: Influenza virus receptors in the human airway. Nature. 2006;440:435–6. Epub 22 March 2006. doi: 10.1038/440435a 16554799 10.1038/440435a

[6] Viswanathan K, Chandrasekaran A, Srinivasan A, Raman R, Sasisekharan V, Sasisekharan R. Glycans as receptors for influenza pathogenesis. Glycoconjugate journal. 2010;27(6):561–70. doi: 10.1007/s10719-010-9303-4 ; PubMed Central PMCID: PMC3407351. 20734133 10.1007/s10719-010-9303-4 PMC3407351

[7] Tumpey TM, Maines TR, Van Hoeven N, Glaser L, Solorzano A, Pappas C, et al A two-amino acid change in the hemagglutinin of the 1918 influenza virus abolishes transmission. Science. 2007;315(5812):655–9. doi: 10.1126/science.1136212 . 17272724 10.1126/science.1136212

[8] Stevens J, Blixt O, Tumpey TM, Taubenberger JK, Paulson JC, Wilson IA. Structure and receptor specificity of the hemagglutinin from an H5N1 influenza virus. Science. 2006;312(5772):404–10. doi: 10.1126/science.1124513 . 16543414 10.1126/science.1124513

[9] Stevens J, Blixt O, Chen LM, Donis RO, Paulson JC, Wilson IA. Recent avian H5N1 viruses exhibit increased propensity for acquiring human receptor specificity. Journal of molecular biology. 2008;381(5):1382–94. doi: 10.1016/j.jmb.2008.04.016 ; PubMed Central PMCID: PMC2519951. 18672252 10.1016/j.jmb.2008.04.016 PMC2519951

[10] Kumari K, Gulati S, Smith DF, Gulati U, Cummings RD, Air GM. Receptor binding specificity of recent human H3N2 influenza viruses. Virology journal. 2007;4:42 doi: 10.1186/1743-422X-4-42 . 17490484 10.1186/1743-422X-4-42 PMC1876801

[11] Gulati S, Smith DF, Air GM. Deletions of neuraminidase and resistance to oseltamivir may be a consequence of restricted receptor specificity in recent H3N2 influenza viruses. Virology journal. 2009;6:22 doi: 10.1186/1743-422X-6-22 ; PubMed Central PMCID: PMC2649058. 19216793 10.1186/1743-422X-6-22 PMC2649058

[12] Gulati S, Smith DF, Cummings RD, Couch RB, Griesemer SB, St George K, et al Human H3N2 Influenza Viruses Isolated from 1968 To 2012 Show Varying Preference for Receptor Substructures with No Apparent Consequences for Disease or Spread. PloS one. 2013;8(6):e66325 doi: 10.1371/journal.pone.0066325 ; PubMed Central PMCID: PMC3689742. 23805213 10.1371/journal.pone.0066325 PMC3689742

[13] Wang YF, Chang CF, Chi CY, Wang HC, Wang JR, Su IJ. Characterization of glycan binding specificities of influenza B viruses with correlation with hemagglutinin genotypes and clinical features. Journal of medical virology. 2012;84(4):679–85. doi: 10.1002/jmv.23219 . 22337309 10.1002/jmv.23219

[14] Tsai HP, Wang HC, Kiang D, Huang SW, Kuo PH, Liu CC, et al Increasing appearance of reassortant influenza B virus in Taiwan from 2002 to 2005. Journal of clinical microbiology. 2006;44(8):2705–13. Epub 2006/08/08. doi: 10.1128/JCM.02694-05 ; PubMed Central PMCID: PMC1594622. 16891481 10.1128/JCM.02694-05 PMC1594622

[15] Goldstein MA, Tauraso NM. Effect of formalin, beta-propiolactone, merthiolate, and ultraviolet light upon influenza virus infectivity chicken cell agglutination, hemagglutination, and antigenicity. Appl Microbiol. 1970;19(2):290–4. . 5437304 10.1128/am.19.2.290-294.1970 PMC376670

[16] Chang CF, Pan JF, Lin CN, Wu IL, Wong CH, Lin CH. Rapid characterization of sugar-binding specificity by in-solution proximity binding with photosensitizers. Glycobiology. 2011;21(7):895–902. Epub 2011/02/18. doi: 10.1093/glycob/cwr021 . 21325337 10.1093/glycob/cwr021

[17] Wang SF, Lee YM, Chan YJ, Liu HF, Yen YF, Liu WT, et al Influenza A virus in Taiwan, 1980–2006: Phylogenetic and antigenic characteristics of the hemagglutinin gene. Journal of medical virology. 2009;81(8):1457–70. doi: 10.1002/jmv.21531 . 19551820 10.1002/jmv.21531 PMC7166446

[18] Rogers GN, Paulson JC, Daniels RS, Skehel JJ, Wilson IA, Wiley DC. Single amino acid substitutions in influenza haemagglutinin change receptor binding specificity. Nature. 1983;304(5921):76–8. Epub 1983/07/07. . 6191220 10.1038/304076a0

[19] Lin YP, Xiong X, Wharton SA, Martin SR, Coombs PJ, Vachieri SG, et al Evolution of the receptor binding properties of the influenza A(H3N2) hemagglutinin. Proceedings of the National Academy of Sciences of the United States of America. 2012;109(52):21474–9. Epub 2012/12/14. doi: 10.1073/pnas.1218841110 ; PubMed Central PMCID: PMC3535595. 23236176 10.1073/pnas.1218841110 PMC3535595

[20] Stohr K. Influenza—WHO cares. The Lancet Infectious diseases. 2002;2(9):517 . 12206966 10.1016/s1473-3099(02)00366-3

[21] WHO. Fact sheet Number 211 Influenza. 2003;http://www.who.int/mediacentre/factsheets/fs211.

[22] Akazawa M, Sindelar JL, Paltiel AD. Economic costs of influenza-related work absenteeism. Value in health: the journal of the International Society for Pharmacoeconomics and Outcomes Research. 2003;6(2):107–15. doi: 10.1046/j.1524-4733.2003.00209.x . 12641861 10.1046/j.1524-4733.2003.00209.x

[23] Rambaut A, Pybus OG, Nelson MI, Viboud C, Taubenberger JK, Holmes EC. The genomic and epidemiological dynamics of human influenza A virus. Nature. 2008;453(7195):615–9. doi: 10.1038/nature06945 ; PubMed Central PMCID: PMC2441973. 18418375 10.1038/nature06945 PMC2441973

[24] Meisen I, Dzudzek T, Ehrhardt C, Ludwig S, Mormann M, Rosenbruck R, et al The human H3N2 influenza viruses A/Victoria/3/75 and A/Hiroshima/52/2005 preferentially bind to alpha2-3-sialylated monosialogangliosides with fucosylated poly-N-acetyllactosaminyl chains. Glycobiology. 2012;22(8):1055–76. doi: 10.1093/glycob/cws077 . 22534568 10.1093/glycob/cws077

[25] Stevens J, Chen LM, Carney PJ, Garten R, Foust A, Le J, et al Receptor specificity of influenza A H3N2 viruses isolated in mammalian cells and embryonated chicken eggs. Journal of virology. 2010;84(16):8287–99. doi: 10.1128/JVI.00058-10 ; PubMed Central PMCID: PMC2916524. 20519409 10.1128/JVI.00058-10 PMC2916524

[26] Medeiros R, Escriou N, Naffakh N, Manuguerra JC, van der Werf S. Hemagglutinin residues of recent human A(H3N2) influenza viruses that contribute to the inability to agglutinate chicken erythrocytes. Virology. 2001;289(1):74–85. Epub 2001/10/17. doi: 10.1006/viro.2001.1121 . 11601919 10.1006/viro.2001.1121

[27] Asaoka N, Tanaka Y, Sakai T, Fujii Y, Ohuchi R, Ohuchi M. Low growth ability of recent influenza clinical isolates in MDCK cells is due to their low receptor binding affinities. Microbes and infection / Institut Pasteur. 2006;8(2):511–9. Epub 2005/11/23. doi: 10.1016/j.micinf.2005.08.006 . 16300986 10.1016/j.micinf.2005.08.006

[28] Air GM. Influenza virus-glycan interactions. Current opinion in virology. 2014;7:128–33. doi: 10.1016/j.coviro.2014.06.004 ; PubMed Central PMCID: PMC4149921. 25061947 10.1016/j.coviro.2014.06.004 PMC4149921

[29] Chandrasekaran A, Srinivasan A, Raman R, Viswanathan K, Raguram S, Tumpey TM, et al Glycan topology determines human adaptation of avian H5N1 virus hemagglutinin. Nature biotechnology. 2008;26(1):107–13. doi: 10.1038/nbt1375 . 18176555 10.1038/nbt1375

[30] Webster RG. Antigenic variation in influenza viruses, with special reference to Hong Kong influenza. Bulletin of the World Health Organization. 1969;41(3):483–5. ; PubMed Central PMCID: PMC2427705. 5309459 PMC2427705

[31] Desselberger U, Nakajima K, Alfino P, Pedersen FS, Haseltine WA, Hannoun C, et al Biochemical evidence that "new" influenza virus strains in nature may arise by recombination (reassortment). Proceedings of the National Academy of Sciences of the United States of America. 1978;75(7):3341–5. ; PubMed Central PMCID: PMC392771. 277933 10.1073/pnas.75.7.3341 PMC392771

[32] Schild GC, Oxford JS, Dowdle WR, Coleman M, Pereira MS, Chakraverty P. Antigenic variation in current influenza A viruses: evidence for a high frequency of antigenic 'drift' for the Hong Kong virus. Bulletin of the World Health Organization. 1974;51(1):1–11. ; PubMed Central PMCID: PMC2366252. 4218138 PMC2366252

[33] Stray SJ, Pittman LB. Subtype- and antigenic site-specific differences in biophysical influences on evolution of influenza virus hemagglutinin. Virology journal. 2012;9:91 doi: 10.1186/1743-422X-9-91 ; PubMed Central PMCID: PMC3499391. 22569196 10.1186/1743-422X-9-91 PMC3499391

[34] Webster RG. Predictions for future human influenza pandemics. J Infect Dis. 1997;176 Suppl 1:S14–9. . 9240688 10.1086/514168

