# Supplementary material for: Glycan-binding preferences and genetic evolution of human seasonal influenza A(H3N2) viruses during 1999-2007 in Taiwan
Source: PLoS One. 2018 May 10;13(5):e0196727. doi: 10.1371/journal.pone.0196727 (PMC5945028; doi:10.1371/journal.pone.0196727)
Supplement: S1 Table — (PDF) [file pone.0196727.s001.pdf]

S1 Table. The list of the influenza viruses accession numbers used in this study.

Virus strain	accession number	Virus strain	accession number
A/Taiwan/N929/1999	KU662128	A/ Taiwan/8/2002	EU501184
A/Taiwan/N467/1999	KU662129	A/Taiwan/TW-1521/2002	EU514674
A/Taiwan/1537/1999	AF362813	A/Taiwan/TW-1522/2002	EU514676
A/Taiwan/1008/1999	AF362812	A/Taiwan/478/2002	KC738694
A/Taiwan/0830/1999	AF362811	A/Taiwan/299/2002	KC738693
A/Taiwan/0389/1999	AF362808	A/Taiwan/231/2002	KC738692
A/Taiwan/3460/2000	AF362819	A/Taiwan/92/2002	KC738691
A/Taiwan/3083/2000	AF362818	A/Taiwan/4954/2002	AY604815
A/Taiwan/0149/2000	AF362806	A/Taiwan/5153/2002	AY604816
A/Taiwan/3760/2000	AF362804	A/Taiwan/3744/2002	AY604814
A/Taiwan/N668/2000	KU662125	A/Taiwan/4673/2002	AY604813
A/Taiwan/N727/2000	KU662126	A/Taiwan/4680/2002	AY604812
A/Taiwan/M822/2000	KU662127	A/Taiwan/3131/2002	AY604811
A/Taiwan/N402/2000	MG309870	A/Taiwan/4963/2002	AY604810
A/Taiwan/N349/2000	MG309871	A/Taiwan/4938/2002	AY604809
A/Taiwan/N1349/2000	MG309872	A/Taiwan/S41/2003	MG309860
A/Taiwan/384/2001	KC738690	A/Taiwan/H0058/2003	MG309861
A/Taiwan/160/2001	KC738689	A/Taiwan/N0741/2003	MG309862
A/Taiwan/124/2001	KC738688	A/Taiwan/N1773/2003	MG309863
A/Taiwan/55/2001	KC738687	A/Taiwan/M0033/2003	MG309864
A/Taiwan/0568/2001	AY625730	A/Taiwan/M195/2003	MG309865
A/Taiwan/0964/2001	AY625731	A/Taiwan/N874/2003	MG309866
A/Taiwan/0388/2001	AY625729	A/Taiwan/M195/2003	KU662113
A/Taiwan/92605/2001	KP458785	A/Taiwan/N123/2003	KU662114
A/Taiwan/1/2001	KP456982	A/Taiwan/N163/2003	KU662115
A/Taiwan/22104/2001	KP456466	A/Taiwan/TW-1530/2003	EU502349
A/Taiwan/N225/2001	KU662122	A/Taiwan/TW-1526/2003	EU502348
A/Taiwan/N615/2001	KU662123	A/Taiwan/1529/2003	EU501334
A/Taiwan/N934/2001	KU662124	A/Taiwan/144/2003	KC738712
A/Taiwan/M063/2002	KU662118	A/Taiwan/89/2003	KC738708
A/Taiwan/N1811/2002	KU662119	A/Taiwan/78/2003	KC738707
A/Taiwan/N2205/2002	KU662120	A/Taiwan/1921/2003	EU068155
A/Taiwan/N4069/2002	KU662121	A/Taiwan/1904/2003	EU068154
A/Taiwan/M190/2002	MG309867	A/Taiwan/3075/2003	EU068152
A/Taiwan/S50/2002	MG309868	A/Taiwan/7196/2003	EU068150
A/Taiwan/S0089/2002	MG309869	A/Taiwan/1533/2003	EU068119

A/Taiwan/5/2003	EU068118	A/Taiwan/S164/2005	MG309832
A/Taiwan/4548/2003	EU068114	A/Taiwan/M152/2005	MG309833
A/Taiwan/158/2003	EU068116	A/Taiwan/N774/2005	MG309834
A/Taiwan/1613/2003	EU068117	A/Taiwan/S99/2005	MG309835
A/Taiwan/N264/2004	KU662109	A/Taiwan/M51/2005	MG309836
A/Taiwan/N1620/2004	KU662110	A/Taiwan/M249/2005	MG309837
A/Taiwan/M687/2004	KU662111	A/Taiwan/M453/2005	MG309838
A/Taiwan/S337/2004	KU662112	A/Taiwan/N2596/2005	MG309839
A/Taiwan/N1496/2004	MG309847	A/Taiwan/S158/2005	MG309840
A/Taiwan/N2173/2004	MG309848	A/Taiwan/N3120/2005	MG309841
A/Taiwan/N784/2004	MG309849	A/Taiwan/S561/2005	MG309842
A/Taiwan/S250/2004	MG309850	A/Taiwan/M352/2005	MG309843
A/Taiwan/M564/2004	MG309851	A/Taiwan/S581/2005	MG309844
A/Taiwan/N2716/2004	MG309852	A/Taiwan/N3059/2005	MG309845
A/Taiwan/N2767/2004	MG309853	A/Taiwan/N1027/2005	MG309846
A/Taiwan/N3161/2004	MG309854	A/Taiwan/N1306/2005	KU662105
A/Taiwan/N3568/2004	MG309855	A/Taiwan/N1801/2005	KU662106
A/Taiwan/N2784/2004	MG309856	A/Taiwan/N2573/2005	KU662107
A/Taiwan/N305/2004	MG309857	A/Taiwan/N2952/2005	KU662108
A/Taiwan/M18/2004	MG309858	A/Taiwan/7601/2005	EU068144
A/Taiwan/H0007/2004	MG309859	A/Taiwan/4865/2005	EU068143
A/Taiwan/1511/2004	EU068153	A/Taiwan/4829/2005	EU068142
A/Taiwan/592/2004	EU068151	A/Taiwan/3294/2005	EU068133
A/Taiwan/3008/2004	EU068149	A/Taiwan/3387/2005	EU068132
A/Taiwan/1651/2004	EU068148	A/Taiwan/4836/2005	EU068130
A/Taiwan/587/2004	EU068147	A/Taiwan/7873/2005	EU068129
A/Taiwan/556/2004	EU068146	A/Taiwan/5267/2005	EU068128
A/Taiwan/3154/2004	EU068145	A/Taiwan/4990/2005	EU068127
A/Taiwan/3187/2004	EU068141	A/Taiwan/5694/2005	EU068126
A/Taiwan/93/2004	EU068140	A/Taiwan/1315/2005	EU068125
A/Taiwan/1817/2004	EU068139	A/Taiwan/7681/2005	EU068124
A/Taiwan/1219/2004	EU068138	A/Taiwan/7702/2005	EU068123
A/Taiwan/7568/2004	EU068135	A/Taiwan/4987/2005	EU068122
A/Taiwan/3245/2004	EU068134	A/Taiwan/4883/2005	EU068121
A/Taiwan/4735/2004	EU068136	A/Taiwan/268/2005	EU068120
A/Taiwan/41/2004	EU068137	A/Taiwan/53/2005	EU501785
A/Taiwan/N509/2005	MG309831	A/Taiwan/431/2005	FJ805517
A/Taiwan/260/2005	FJ805513	A/Taiwan/N1068/2007	MG309823

A/Taiwan/601/2005	KC738760	A/Taiwan/N59/2007	MG309824
A/Taiwan/N763/2006	MG309828	A/Taiwan/M47/2007	MG309825
A/Taiwan/N3078/2006	MG309829	A/Taiwan/N1019/2007	MG309826
A/Taiwan/M234/2006	MG309830	A/Taiwan/N1215/2007	MG309827
A/Taiwan/M602/2006	KU662101	A/Taiwan/M147/2007	KU662097
A/Taiwan/N1558/2006	KU662102	A/Taiwan/M183/2007	KU662098
A/Taiwan/N2560/2006	KU662103	A/Taiwan/N607/2007	KU662099
A/Taiwan/N519/2006	KU662104	A/Taiwan/N661/2007	KU662100
A/Taiwan/2072/2006	EU068131	A/Taiwan/1/2007	KC738783
A/Taiwan/784/2006	EU502062	A/Taiwan/449/2007	FJ805533
A/Taiwan/760/2006	EU502061	A/Taiwan/448/2007	FJ805532
A/Taiwan/99/2006	EU502060	A/Taiwan/216/2007	FJ805531
A/Taiwan/83/2006	EU502059	A/Taiwan/214/2007	FJ805530
A/Taiwan/40/2006	EU502058	A/Taiwan/132/2007	FJ805529
A/Taiwan/799/2006	FJ805524	A/Taiwan/33/2007	FJ805528
A/Taiwan/758/2006	FJ805522	A/Taiwan/18/2007	FJ805527
A/Taiwan/754/2006	FJ805521	A/Taiwan/7/2007	FJ805526
A/Taiwan/211/2006	FJ805520	A/Taiwan/5/2007	FJ805525
A/Taiwan/133/2006	FJ805519	A/Taiwan/306/2007	KC738804
A/Taiwan/17/2006	FJ805518	A/Taiwan/304/2007	KC738803
A/Taiwan/729/2006	KC738775	A/Taiwan/303/2007	KC738793
A/Taiwan/645/2006	KC738774	A/Taiwan/131/2007	KC738792
A/Taiwan/15/2006	KC738773	A/Taiwan/302/2007	KC738791
A/Taiwan/1/2006	KC738772	A/Taiwan/301/2007	KC738790
A/Taiwan/788/2006	KC738771	A/Taiwan/212/2007	KC738789
A/Taiwan/776/2006	KC738770	A/Taiwan/103/2007	KC738788
A/Taiwan/718/2006	KC738769	A/Taiwan/102/2007	KC738787
A/Taiwan/717/2006	KC738768	A/Taiwan/57/2007	KC738786
A/Taiwan/100/2006	KC738767	A/Taiwan/19/2007	KC738785
A/Taiwan/N263/2007	MG309818	A/Taiwan/3/2007	KC738784
A/Taiwan/N542/2007	MG309819		
A/Taiwan/M146/2007	MG309820		
A/Taiwan/N334/2007	MG309821		
A/Taiwan/N483/2007	MG309822		
